# High-throughput sequencing reveals the virome characteristics of wastewater in Gansu Province, China from 2024 to 2026

**DOI:** 10.3389/fmicb.2026.1966428

**Published:** 2026-09-22

**Authors:** Yabei Xu, Xiaohong Zhao, Shang Gao, Jinbo Xiao, Qian Yang, Jingjing Xu, Yuecong Xie, Yixin Xu, Xinghao Ye, Tianjiao Ji, Yong Zhang, Dongmei Yan

**Affiliations:** 1 National Laboratory for Poliomyelitis, WHO WPRO Regional Polio Reference Laboratory, National Key Laboratory of Intelligent Tracking and Forecasting for Infectious Diseases, National Health Commission Key Laboratory of Microbial Genomics, National Institute for Viral Disease Control and Prevention, Chinese Center for Disease Control and Prevention & Chinese Academy of Preventive Medicine, Beijing, China; 2 Gansu Center for Disease Control and Prevention & Gansu Provincial Academy of Preventive Medicine, Lanzhou, China; 3 School of Basic Medicine, Shandong Second Medical University, Weifang, China

**Keywords:** astroviruses, enterovirus, Gansu Province, metagenomic sequencing, targeted next-generation sequencing, wastewater

## Abstract

Emerging and re-emerging infectious diseases continue to pose a persistent challenge to global public health security. As an unbiased and noninvasive population-level surveillance approach, wastewater-based epidemiology has emerged as an urban-scale monitoring tool for proactively capturing the community disease burden. In this study, metagenomic and targeted next-generation sequencing were employed to preliminarily characterize wastewater viral communities in Gansu Province from 2024 to 2026. Across the three annual sampling groups, the Simpson diversity index calculated from metagenomic taxonomic profiles showed a significant negative correlation with sampling year. Despite viral enrichment, more than 97% of sequencing reads were assigned to bacteria, likely reflecting residual nonviral nucleic acids and limitations in metagenomic classification. The wastewater virome exhibited a complex host-association spectrum, within which bacteriophages and plant-associated viruses displayed relatively stable community structures and high relative abundances. Among human pathogens, astroviruses were consistently detected at high relative abundances across all samples. Human astrovirus 1 accounted for the largest proportion, and a nearly complete genome sequence of the extraintestinal infection-associated astrovirus MLB2 was recovered. Targeted sequencing further revealed a highly structured environmental enterovirus profile dominated by the species *Enterovirus betacoxsackie*, followed by *Enterovirus alphacoxsackie*, whereas *Enterovirus coxsackiepol* was detected at an extremely low abundance and *Enterovirus deconjuncti* was not detected. In addition to several common viruses associated with hand, foot, and mouth disease, rare viruses, including coxsackievirus A19 and enterovirus B83, were successfully captured. Echovirus 11 was identified as the predominant enterovirus serotype in current wastewater surveillance in Gansu Province. In summary, this study provides a preliminary characterization of the viral community profiles in wastewater samples from Gansu Province, demonstrates the effectiveness of wastewater-based epidemiology for population-level surveillance, and underscores the need to establish a coordinated defense network integrating clinical case surveillance with environmental monitoring.

## Introduction

Wastewater-based epidemiology has become a prominent approach to noninvasive public health surveillance, offering a comprehensive snapshot of community-level infectious disease dynamics and environmental health (Crits-Christoph et al., 2021). Driven by advances in high-throughput sequencing, the field has successfully transitioned from targeted PCR assays to untargeted, whole-community metagenomic and metatranscriptomic profiling (Tan et al., 2015). Within municipal wastewater, the virome serves as a critical reservoir of biological information, granting real-time insights into the circulating viruses—including human pathogens, bacteriophages, and diverse environmental viruses—thereby acting as a crucial early warning system for emerging infectious diseases (Rothman et al., 2021). Therefore, profiling of the wastewater virome is imperative for comprehensively assessing viral ecological patterns and population health.

Gansu Province, located in northwestern China, serves as an ecologically significant region characterized by a distinct arid climate and unique sociogeographical attributes. Although the region’s resident population reached 24.43 million by the end of 2025, Gansu remains a largely unexplored territory in environmental microbiology despite its profound socioenvironmental significance. To bridge this spatial knowledge gap, this study conducted multiyear wastewater sampling across 6 representative cities. Specifically, raw wastewater samples were collected from wastewater treatment plants in Lanzhou, the provincial capital characterized by high population mobility; Hezuo, which has a multiethnic population; Zhangye, an important transportation hub; Jiuquan and Longnan, which border the Xinjiang Uygur Autonomous Region and Sichuan Province, respectively; and Dingxi, located in central Gansu. The catchment populations served by the sampled wastewater treatment plants exceeded 1 million in Lanzhou and Longnan and 100,000 at each of the other sites. Using high-throughput sequencing, we conducted an exploratory characterization of wastewater viral signals across selected cities in Gansu Province between 2024 and 2026, providing preliminary evidence to support local public health surveillance and targeted decision-making.

## Materials and methods

### Collection and processing of the wastewater samples

Between 2024 and 2026, 18 raw wastewater samples were collected from wastewater treatment plants across 6 cities in Gansu Province (Hezuo, Zhangye, Lanzhou, Jiuquan, Longnan, and Dingxi). Based on their respective sampling years, the samples were categorized into 3 groups: GS24, GS25, and GS26. Seasonal patterns, population differences, and other demographic factors were not considered when defining these groups. Specifically, the sample groups were designated as follows: GS24-1 to GS24-6 were collected in July 2024 from Hezuo, Zhangye, Lanzhou, Jiuquan, Longnan, and Hezuo, respectively; GS25-1 to GS25-6 were collected in June 2025 from Hezuo, Zhangye, Lanzhou, Jiuquan, Longnan, and Zhangye, respectively; and GS26-1 to GS26-6 were collected in March 2026 from Hezuo, Zhangye, Lanzhou, Jiuquan, Longnan, and Dingxi, respectively. GS24-1 and GS24-6 were independently collected on the same day from the same wastewater treatment plant in Hezuo. The same applied to GS25-2 and GS25-6 in Zhangye. The collection dates reflected operational availability and were not selected to provide seasonally matched observations. At each monitoring site, 1 L of raw wastewater was collected. The samples were transported at 4 °C to the laboratory of Gansu Provincial Center for Disease Control and Prevention within 7 days and processed immediately upon arrival (Wang et al., 2014). For viral concentration, the wastewater samples were initially centrifuged at 1,000 × *g* for 30 min at 4 °C. Subsequently, 2.5 M MgCl_2_ was added to the supernatant to achieve a final concentration of 0.05 M, and the pH was adjusted to 3.5 using 0.5 N HCl. The prepared solution was then filtered through a 142-mm cellulose nitrate membrane using a positive-pressure pump. Following filtration, the membrane was fragmented and eluted twice with 10 mL of 3% beef extract by vortexing on ice for 5 min each time. The pooled eluates were centrifuged at 1,000 × *g* for 15 min at 4 °C and finally passed through a 0.22 μm syringe filter.

### Nucleic acid extraction and sequencing

Total nucleic acid extraction was performed using the High Pure Viral Nucleic Acid Kit (Roche) without DNase digestion. To reduce potential false-positive results associated with the increased PCR cycles required for capture-based sequencing, the initial extract was divided for parallel and independent library preparations. Metagenomic libraries were generated with the MGIEasy Fast RNA Library Prep Set, whereas the IGT® Fast Stranded RNA Library Prep Kit v2.0 was applied for capture-based construction. For targeted enrichment, a custom enterovirus probe panel was deployed; this panel was specifically designed for selected viruses, encompassing the species *Enterovirus alphacoxsackie*, *Enterovirus betacoxsackie*, *Enterovirus coxsackiepol*, and *Enterovirus deconjuncti*. Negative controls were processed in parallel during nucleic acid extraction and library preparation to monitor potential contamination, and no evidence of contamination or other procedural abnormalities was observed. Finally, paired-end reads were generated by sequencing the prepared libraries on a DNBSEQ-T7 platform.

### Sequence data analysis

Raw sequencing reads generated by the metagenomic and targeted-capture workflows were assessed using FastQC^1^ before downstream analysis. No sequencing dataset was excluded from downstream analysis solely on the basis of the FastQC assessment. For raw paired-end reads, adapter trimming and quality filtering (length <50 bp, quality score <20, or containing N bases) were performed using KneadData.^2^ High-quality reads were then aligned to the human reference genome (hg19) via Bowtie2 to discard host sequences, with the unmapped reads kept for subsequent analyses (Langmead and Salzberg, 2012). Metagenomic reads were taxonomically classified using Kraken2 across multiple taxonomic levels (Wood et al., 2019). All sequencing reads were assembled using MEGAHIT (Li et al., 2015). Contigs (length ≥300 bp, coverage ≥3×) were filtered via awk and annotated using blastn, taking the highest bitscore as the final assignment (Chen et al., 2015). Read abundances per viral species were quantified using BWA-backtrack for downstream evaluation (Li and Durbin, 2009). All astrovirus- and enterovirus-associated contigs assembled using MEGAHIT were extracted from each sample and individually re-evaluated using NCBI BLASTn.^3^

### Rarefaction analysis and diversity visualization

All statistical analyses and data visualizations were performed using R 4.4.3 software.^4^ Specifically, alpha diversity was assessed at the genus level, and the Shannon and inverse Simpson indices were calculated using the vegan package. Hereafter, the term “Simpson index” refers specifically to the inverse Simpson formulation (1/*D*). Temporal trends in alpha diversity were assessed using one-tailed Spearman’s rank correlation tests, with the *a priori* hypothesis that diversity indices would decline over sampling years. Spearman’s rho (*ρ*) values and corresponding one-sided *p* values were calculated to evaluate the monotonic relationship between sampling year and alpha diversity indices. Rarefaction curves, heatmaps, stacked bar plots, and visualizations of alpha diversity indices were generated using the ggplot2 package.

### Quantitative profiling of the virome

For the quantitative analysis of viral communities, relative abundances were estimated using the reads per million normalization method described in Equation 1. The mapped read counts of each viral contig were adjusted for genome length differences by dividing the read counts by the average genome size of the corresponding viral family or genus. Subsequently, the adjusted read counts were normalized against the total number of reads assigned to all viral families or genera within each individual sample (Worp et al., 2025). This normalization strategy was consistently applied to all heatmaps generated in this study.

However, a modified approach was applied for the stacked bar charts illustrating the relative abundances of different species within the genus *Enterovirus*. Because genome lengths among enterovirus serotypes are highly conserved, with an average size of approximately 7.4 kb, genome length normalization was omitted when calculating the relative abundance of enterovirus-associated contigs.

$$\text{Relative abundance}=\frac{\text{virus reads}}{\text{average genome length}\times \text{total viral reads}}\times 10^{6}$$ (1)

The host type classifications for the viral families listed herein were retrieved from the Virus-Host Database.^5^

### Comparison of viral diversity

To compare viral community structure across sample groups, principal component analysis (PCA) was performed on centered log-ratio transformed abundance data (Gloor et al., 2017). Zero counts in the abundance matrices were replaced with a pseudocount equivalent to half of the minimum positive abundance value detected within the respective dataset, as centered log-ratio transformation requires strictly positive values. Viral taxa contributing most strongly to the principal components were identified based on the magnitude of their loadings. PCA biplots were generated using the ggplot2 package in R 4.4.3 software, with 68% confidence ellipses overlaid to visualize group clustering.

### Phylogenetic analysis

Reference viral VP1 gene sequences were retrieved from GenBank at the National Center for biotechnology information (NCBI). These sequences, together with the VP1 sequences generated in this study, were aligned using MAFFT v7.520 (Katoh and Standley, 2013). Neighbor-joining (NJ) phylogenetic trees were constructed in MEGA7 based on evolutionary distances calculated using the maximum composite likelihood method (Kumar et al., 2016). The robustness of the inferred tree topologies was assessed by bootstrap analysis with 1,000 replicates (Felsenstein, 1985), and bootstrap support values of 70% or higher were displayed at the corresponding nodes.

## Results

### Between-year variation in alpha diversity and broad taxonomic composition

Rarefaction analysis showed that the sequencing captured most of the detectable genus-level diversity across the three sampling groups, indicating that the sequencing depth was sufficient (Figure 1A). Overall metagenomic sequencing statistics and alpha diversity indices (including the Shannon and Simpson indices) are summarized in Table 1 and visualized in Figures 1B,C. Sampling year was treated as an ordinal variable. The Shannon index showed a negative but nonsignificant correlation with sampling year (Spearman’s *ρ* = −0.354, *p* = 0.075). In contrast, the Simpson index exhibited a significant negative correlation with sampling year (Spearman’s *ρ* = −0.472, *p* = 0.024). Given that the dataset comprised only three annual sampling campaigns conducted in different months, these analyses were considered exploratory assessments of between-year variation. At the broadest taxonomic level, more than 97% of reads in each dataset were assigned to Bacteria, whereas no more than 0.94% were classified as viral (Table 2). Following taxonomic classification with Kraken2, 56.42–74.74% of the metagenomic reads across samples remained unclassified at the genus level (Supplementary Table S1). In this context, “unclassified” indicates that the reference database and parameter settings did not provide sufficient evidence for a reliable taxonomic assignment. The unclassified reads were not subjected to further characterization in this study.

**Figure 1:**
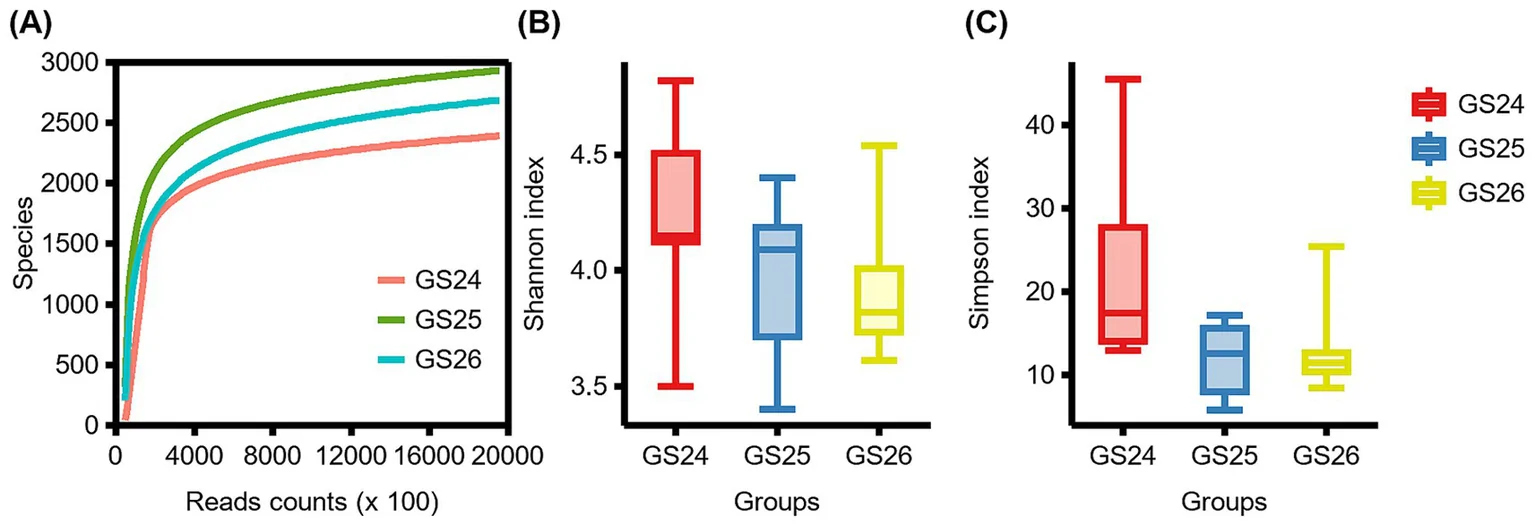
Overview of metagenomic sequencing data across the three groups. **(A)** Rarefaction curves at the genus level. **(B)** Shannon index. **(C)** Simpson index.

**Table 1:** Sequencing statistics and alpha diversity indices of analyzed samples.

Sampling time	Samples	Raw reads	Clean reads	Shannon index	Simpson index
GS24	GS24-1	199,047,474	195,718,638	4.108451	20.08064
	GS24-2	218,726,918	214,116,796	4.81636	45.418168
	GS24-3	160,375,020	155,794,922	4.627284	30.717302
	GS24-4	325,293,894	319,249,082	4.122234	14.731783
	GS24-5	139,015,384	136,263,998	4.188738	12.88284
	GS24-6	322,375,202	315,799,596	3.503961	13.170034
GS25	GS25-1	140,683,592	127,724,692	3.40483	6.452145
	GS25-2	162,592,148	154,692,526	4.394998	13.951328
	GS25-3	158,482,206	148,291,634	3.570504	5.636478
	GS25-4	153,859,314	149,096,490	4.093675	17.063441
	GS25-5	207,868,464	200,949,116	4.237758	16.592169
	GS25-6	163,090,212	154,088,636	4.083157	10.976367
GS26	GS26-1	163,233,600	158,127,660	3.925408	12.987845
	GS26-2	181,330,834	178,565,650	3.72137	9.935286
	GS26-3	252,500,086	247,379,160	3.616099	8.320831
	GS26-4	179,844,562	174,134,254	4.535476	25.35726
	GS26-5	190,260,428	184,138,230	4.042254	12.863528
	GS26-6	170,198,398	161,647,728	3.723048	9.84018

**Table 2:** Proportions of reads assigned to Bacteria, Archaea, Eukaryota, and viruses in each sample at the broadest taxonomic level.

Sampling time	Sample	Bacteria	Archaea	Eukaryota	Viruses
GS24	GS24-1	99.88%	0.08%	0.01%	0.03%
	GS24-2	99.86%	0.07%	0.00%	0.06%
	GS24-3	99.85%	0.07%	0.02%	0.05%
	GS24-4	99.55%	0.24%	0.01%	0.21%
	GS24-5	99.52%	0.16%	0.06%	0.25%
	GS24-6	99.48%	0.15%	0.26%	0.11%
GS25	GS25-1	97.12%	0.43%	2.16%	0.29%
	GS25-2	98.78%	0.40%	0.52%	0.30%
	GS25-3	97.34%	0.34%	1.56%	0.76%
	GS25-4	99.39%	0.19%	0.22%	0.20%
	GS25-5	99.48%	0.15%	0.26%	0.11%
	GS25-6	99.03%	0.50%	0.22%	0.25%
GS26	GS26-1	99.04%	0.14%	0.15%	0.66%
	GS26-2	99.52%	0.16%	0.02%	0.30%
	GS26-3	99.47%	0.25%	0.01%	0.27%
	GS26-4	99.66%	0.15%	0.08%	0.11%
	GS26-5	99.78%	0.16%	0.01%	0.06%
	GS26-6	98.11%	0.31%	0.64%	0.94%

### Prevalence patterns and host associations of viral families of concern

To characterize the viral community structure, we generated a heatmap of representative viral families, revealing a highly diverse multihost virome in the wastewater microecosystem, encompassing archaeal, bacterial, plant, insect, and vertebrate hosts (Figure 2). Specifically, bacteriophages, such as members of the family *Fiersviridae*, and plant-infecting viruses, including members of the family *Alphaflexiviridae*, showed relatively high abundance and consistent detection patterns throughout the study period. In the context of vertebrate hosts, RNA viral families were consistently detected. Notably, the families *Astroviridae* and *Picornaviridae* maintained a stable presence, although a slight decrease in the relative abundance of *Astroviridae* was observed in localized samples from 2025. In contrast, the families *Reoviridae* and *Hepeviridae* consistently remained at low relative abundance across all temporal cohorts.

**Figure 2:**
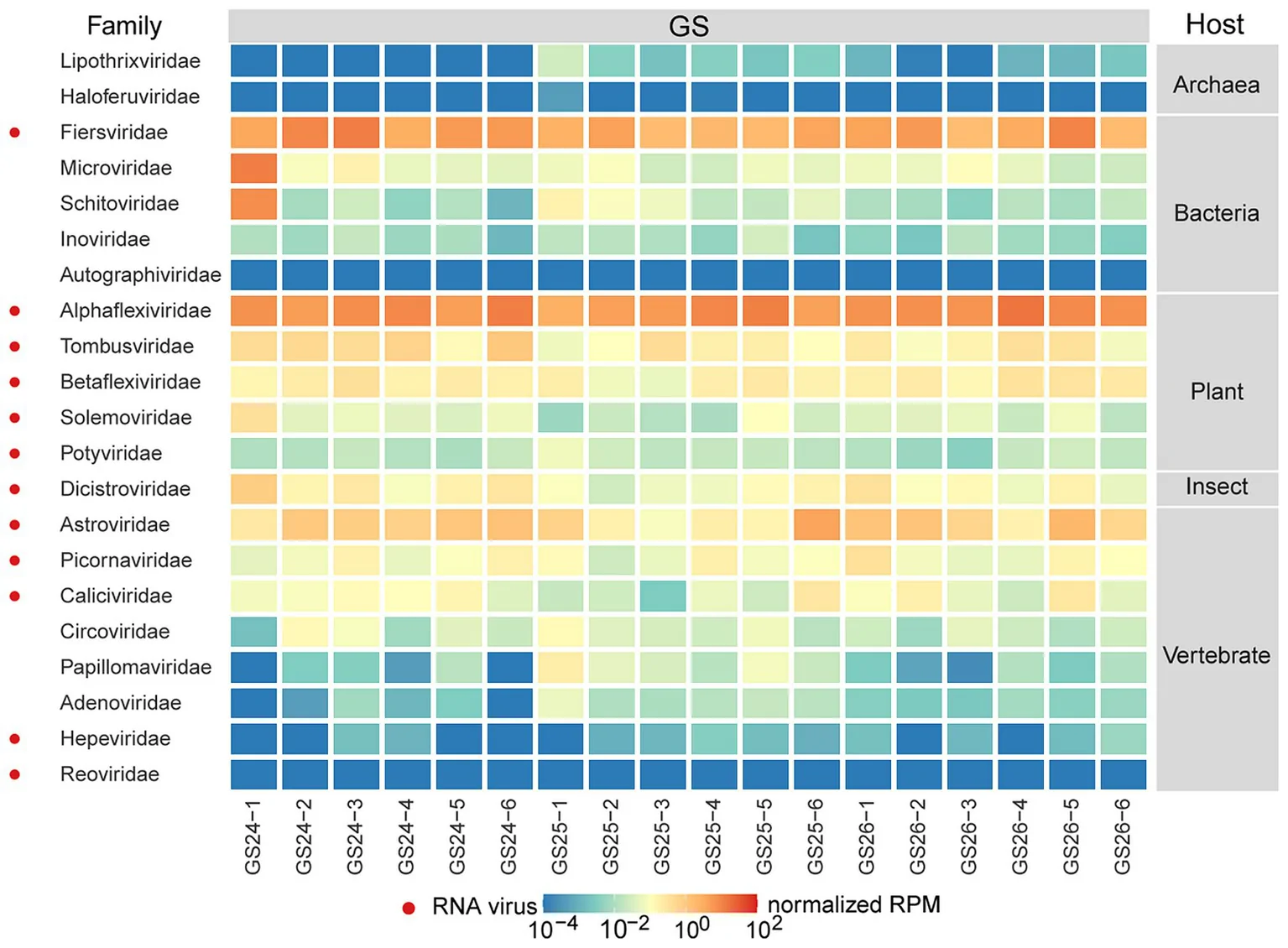
Heatmap of target viral families in Gansu wastewater samples. Viral families are categorized by host, then ranked and color-coded based on their log-transformed, genome-length-normalized relative abundances. RNA viruses are denoted by red dots; DNA viruses remain unmarked.

### Abundance profiles and distribution dynamics of selected viral genera

To characterize the community structure, a relative abundance heatmap at the genus level was constructed by selecting viral genera with high read counts along with 9 clinically relevant viral genera (Figure 3). The analysis revealed a generally stable composition of the wastewater virome. Plant-associated viruses consistently maintained high relative abundances. Aside from this, a highly diverse range of bacteriophages was detected, encompassing genera such as *Carjivirus*, *Nehpavirus*, *Andihavirus*, *Hihdivirus*, *Aldormivirus*, *Plateaulakevirus*, and *Kagunavirus*, alongside insect-infecting viral genera like *Alphanodavirus* and *Betabaculovirus*. The 9 human-associated viral genera prioritized in this study represent viral genera targeting 3 major organ systems: enteric viruses (*Norovirus*, *Rotavirus*, *Enterovirus*, *Mamastrovirus*, *Kobuvirus*, and *Salivirus*), respiratory viruses (*Betacoronavirus* and *Bocaparvovirus*), and liver-targeted viruses (*Hepatovirus*). Among these, *Mamastrovirus* displayed the highest relative abundance, forming a pronounced accumulation peak at the wastewater collection nodes. Excluding *Mamastrovirus*, the relative abundances of the remaining 8 human-associated viral genera were largely comparable and remained at a stable baseline level.

**Figure 3:**
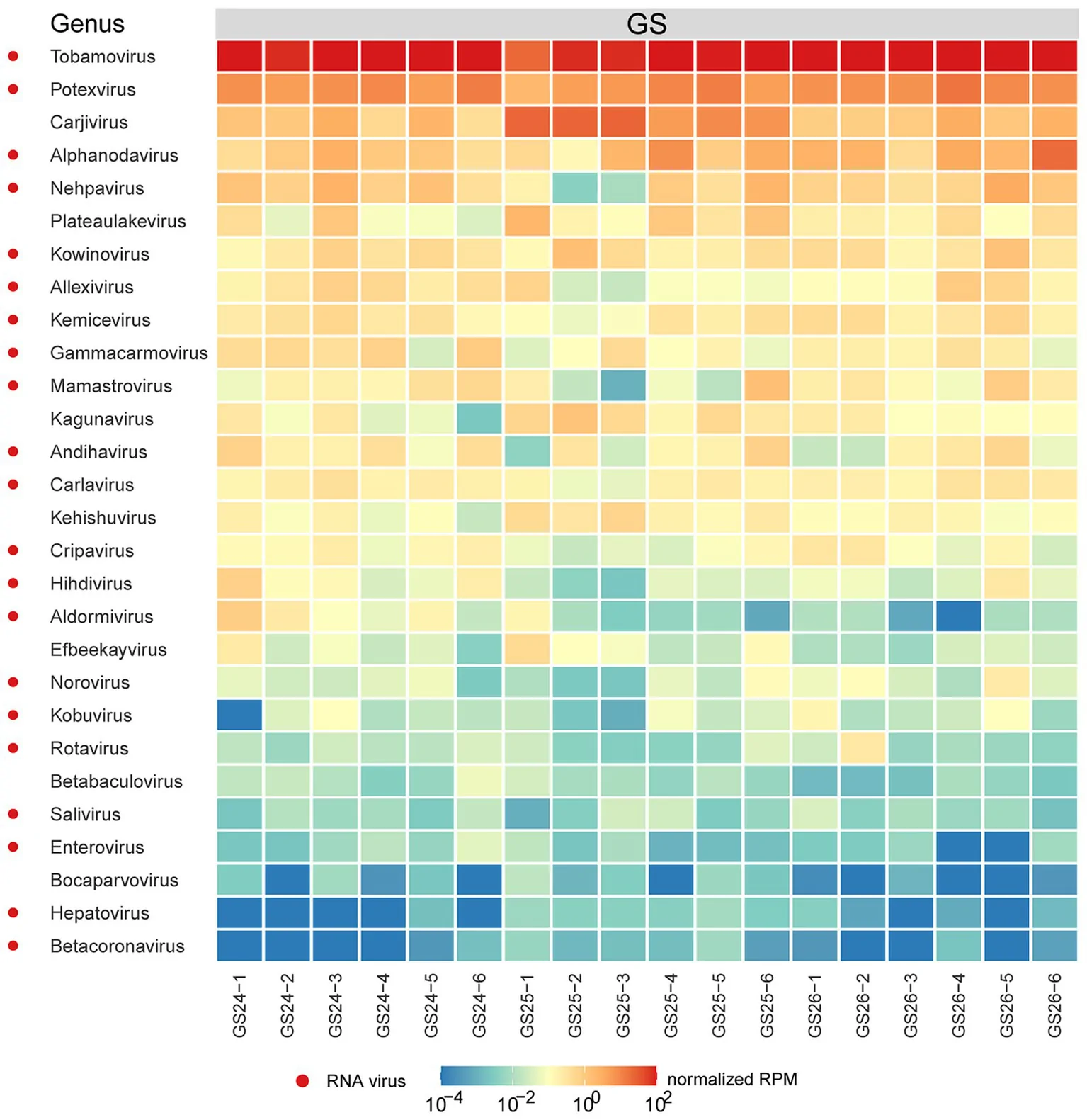
Heatmap of selected viral genera in Gansu wastewater samples. Viral genera are ranked and color-coded based on their log-transformed, genome-length-normalized relative abundances. RNA viruses are denoted by red dots; DNA viruses remain unmarked.

At the selected viral-genus level, PCA showed descriptive separation among the three sampling campaigns (Figure 4). PC1 and PC2 explained 34.0 and 18.1% of the variation, respectively, accounting for 52.1% of the total variation. Because each campaign corresponded to a different collection month, the PCA was considered descriptive and was not used to attribute separation specifically to year.

**Figure 4:**
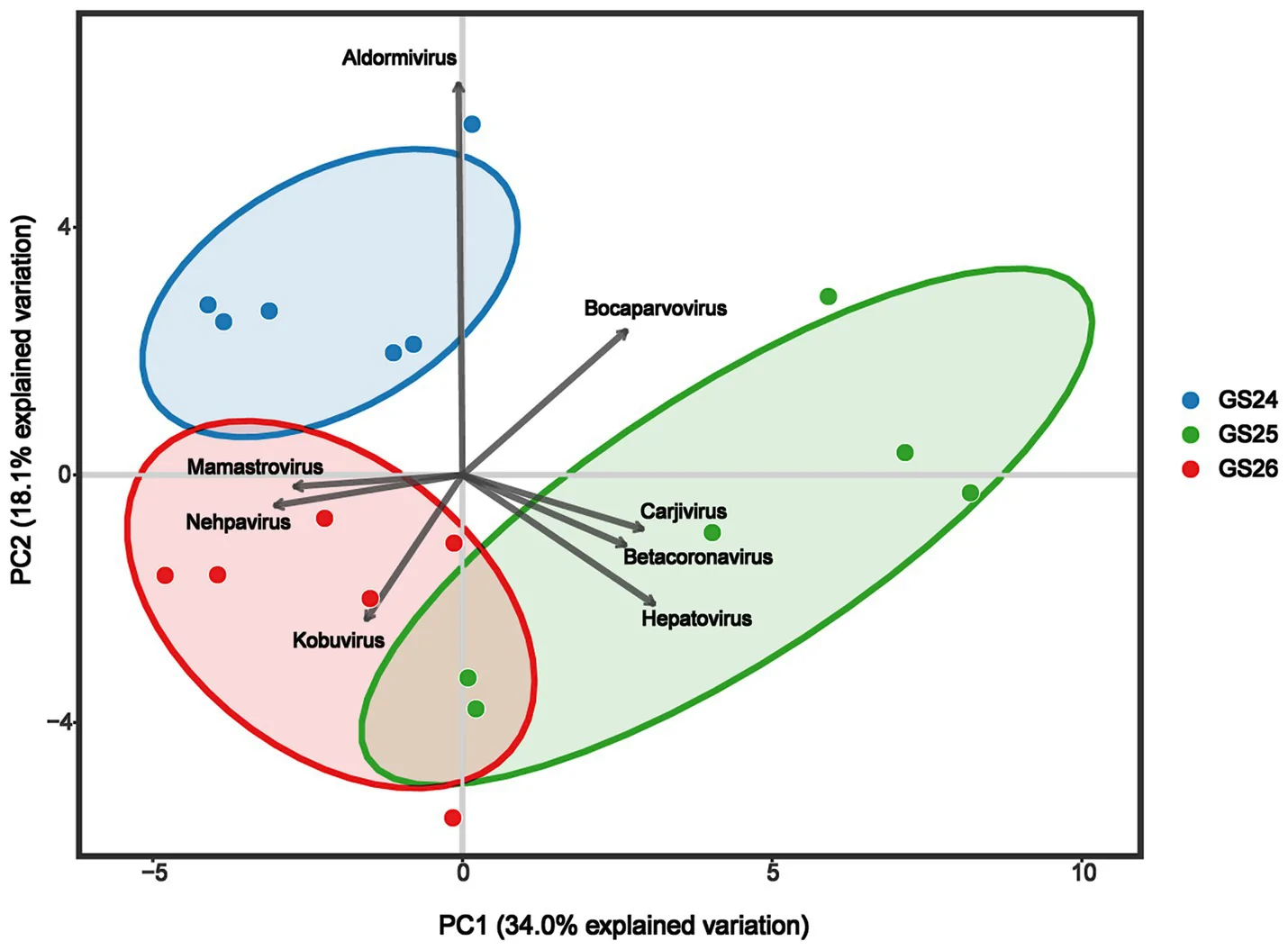
PCA plot based on the composition of selected viral genera in the three groups of wastewater samples. Principal components were calculated from genome-length-normalized read counts following a centered log-ratio transformation. Viral genera are depicted as arrows, with their orientation indicating the direction of their contributions to the principal components and their length representing the magnitude of their loadings.

### Genomic characterization of detected astroviruses

Astroviruses were detected in all 18 wastewater samples. Analysis of the longest assembled contigs for human astrovirus (HAstV) across all samples revealed that classical HAstVs were identified in 15 of the 18 samples, novel HAstVs were detected in 2 samples, with 1 sample being classifiable only as HAstV (Table 3). Both novel HAstV sequences were classified within the HAstV-MLB2 lineage. Among the classical HAstV genotypes, HAstV-1 was predominant, identified in 14 of the 18 samples, followed by HAstV-4 (1/18). Sequence alignment indicated a high nucleotide identity (97.35–99.77%) between the assembled contigs and their respective NCBI reference sequences, with the best-matching strains primarily originating from China (Fujian, Yunnan, and Shandong provinces), Japan, and Thailand. Notably, a near-full-length MLB2 genome sequence (6,018 nt) was assembled from sample GS26-1.

**Table 3:** Characteristics of the longest assembled human astrovirus (HAstV) contigs identified in wastewater samples.

irus identification	Contig ID	Largest contig (nt)	Completeness relative to reference sequence (%)	Best BLASTn hit			
				GB-ID	Strain name	Country	Identity (%)
HAstV-1	GS24-1_k141_2929	454	60.53	OQ543061.1	Human astrovirus 1 isolate CMH-R022-20	Thailand	98.9
HAstV-1	GS24-2_k141_6398	1,686	24.74	PV189389.1	Human astrovirus 1 strain HAstV-1/Fuzhou2023137	China	99.26
HAstV-1	GS24-3_k141_61442	1,642	24.1	PV189386.1	Human astrovirus 1 strain HAstV-1/Fuzhou2023097	China	99.15
HAstV-1	GS24-4_k141_13800	1,677	24.52	LC694995.1	Human astrovirus 1 HAstV1/192642/Tokyo/2020	Japan	97.35
HAstV-1	GS24-5_k141_16088	2045	30.01	PV189386.1	Human astrovirus 1 strain HAstV-1/Fuzhou2023097	China	98.25
HAstV-1	GS24-6_k141_2311	847	12.43	PV189391.1	Human astrovirus 1 strain HAstV-1/Fuzhou2023143	China	99.77
HAstV-1	GS25-1_k141_2945	1,050	15.41	PV189391.1	Human astrovirus 1 strain HAstV-1/Fuzhou2023143	China	98.37
HAstV-1	GS25-2_k141_307868	2,358	34.61	ON571597.1	Human astrovirus 1 isolate 2019/Pingtan/42	China	98.55
Astrovirus MLB2	GS25-3_k141_147856	1,653	26.96	MK327365.1	Astrovirus MLB2 isolate MLB-YJMGK	China	99.05
HAstV-1	GS25-4_k141_46493	717	10.59	OQ968303.1	MAG: Mamastrovirus sp. isolate 20286/SD/CHN/HAstV-1	China	99.47
HAstV	GS25-5_k141_71756	427	6.2	PQ496891.1	Human astrovirus isolate Yunnan_B224	China	99.26
HAstV-4	GS25-6_k141_40212	1,359	20.31	OP432297.1	Human astrovirus 4 isolate HAstV-4/QD-L-22001	China	99.05
Astrovirus MLB2	GS26-1_k141_15392	6,018	98.16	MK327365.1	Astrovirus MLB2 isolate MLB-YJMGK	China	98.73
HAstV-1	GS26-2_k141_18361	2,853	41.88	PV189391.1	Human astrovirus 1 strain HAstV-1/Fuzhou2023143	China	98.77
HAstV-1	GS26-3_k141_22241	1,696	24.89	PV189392.1	Human astrovirus 1 strain HAstV-1/Fuzhou2023194	China	98.19
HAstV-1	GS26-4_k141_61211	399	5.9	PV189391.1	Human astrovirus 1 strain HAstV-1/Fuzhou2023143	China	99.03
HAstV-1	GS26-5_k141_3074	1,542	22.64	PV189391.1	Human astrovirus 1 strain HAstV-1/Fuzhou2023143	China	99.25
HAstV-1	GS26-6_k141_21704	809	11.88	PV189391.1	Human astrovirus 1 strain HAstV-1/Fuzhou2023143	China	99.41

### Relative abundance and phylogenetic analysis of enteroviruses

To further characterize the distribution of enteroviruses (EVs) at the species and type levels in Gansu Province, targeted capture sequencing was performed on the wastewater samples. Targeted next-generation sequencing data uncovered a highly structured ecological hierarchy within the wastewater EV community. At the species level, the EV community profile in the environmental wastewater of Gansu Province was dominated by the species *Enterovirus betacoxsackie*, whereas the relative abundance of the species *Enterovirus alphacoxsackie* exhibited fluctuations. Conversely, *Enterovirus coxsackiepol* was detected at a very low relative abundance, and the species *Enterovirus deconjuncti* was not detected during this surveillance (Figure 5). A total of 26 EV types were detected in wastewater samples, among which enterovirus A71 (EV-A71) and coxsackievirus A16 (CV-A16) were frequently detected, whereas other types, including coxsackievirus A19 and enterovirus B83, were detected less frequently.

**Figure 5:**
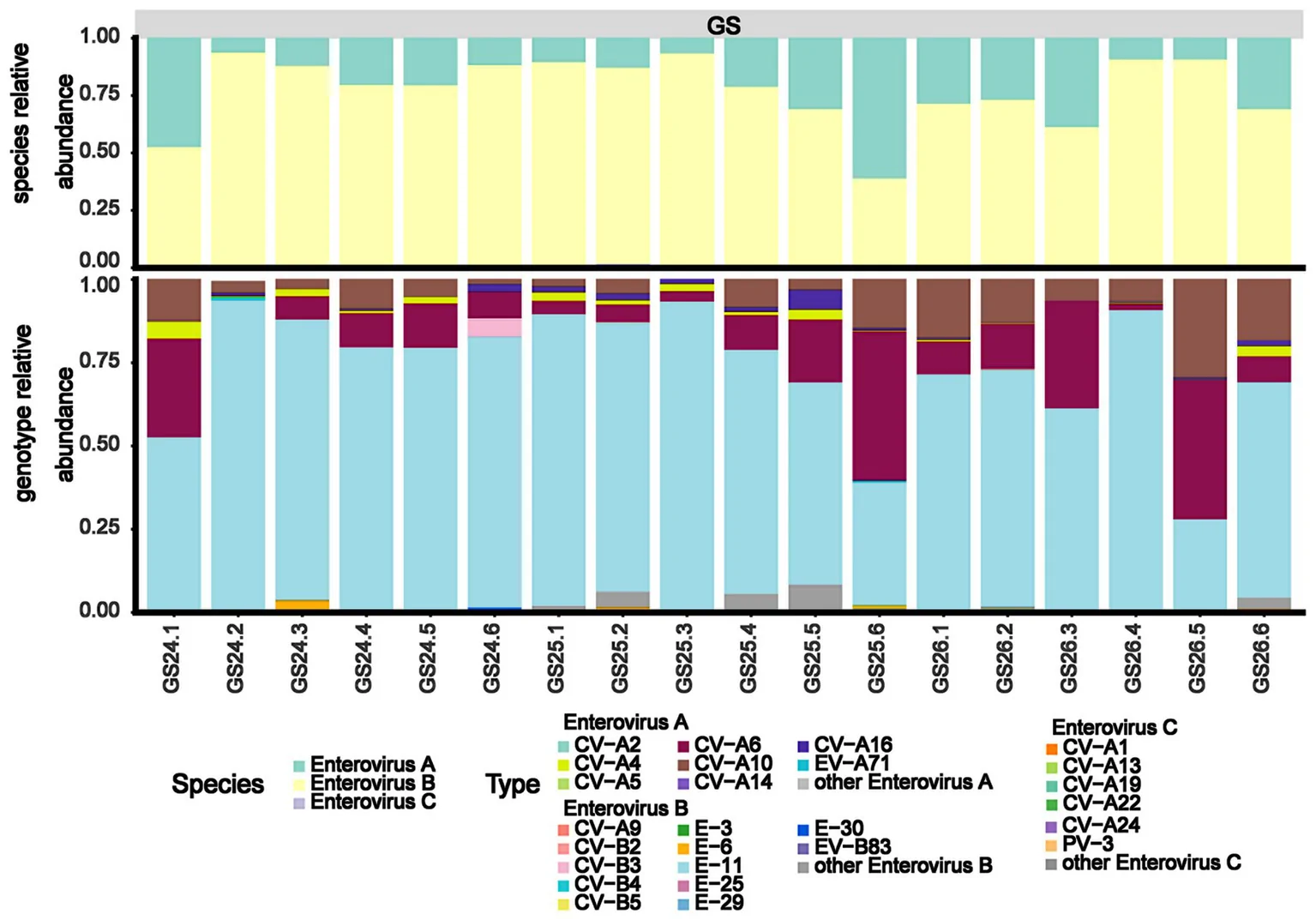
Prevalence and relative abundance of *Enterovirus* species and types in Gansu wastewater based on targeted next-generation sequencing. Stacked bar plots display the relative abundances of *Enterovirus* species and specific types. “Other” represents contigs that could not be assigned to a specific type via BLASTn analysis. The full names of the type abbreviations used in the figure are provided in the Supplementary Table S2.

High-resolution type-level profiling demonstrated that the baseline of the species *Enterovirus betacoxsackie* was primarily anchored by echovirus 11, establishing it as the relatively dominant serotype in current environmental wastewater surveillance. Notably, coxsackievirus B5 and echovirus 30 were also sporadically detected in wastewater. The detection of *Enterovirus alphacoxsackie* was predominantly attributable to coxsackievirus A6 (CV-A6), with concurrent detection of other related serotypes, including CV-A16 and coxsackievirus A10 (CV-A10), all of which are important causative agents of hand, foot, and mouth disease (HFMD).

A contig covering 81.4% of the CV-A16 VP1 coding region was assembled from wastewater sample GS26-6. After trimming, this sequence was aligned with 19 CV-A16 reference sequences representing known genotypes and one EV-A71 sequence (U22521.1) used as the outgroup. Phylogenetic analysis indicated that the CV-A16 sequence recovered in this study clustered with B1b reference strains within the same evolutionary branch, supported by a bootstrap value of 87% at the node, while separating from B1a, B1c, and other genotype reference strains (Figure 6).

**Figure 6:**
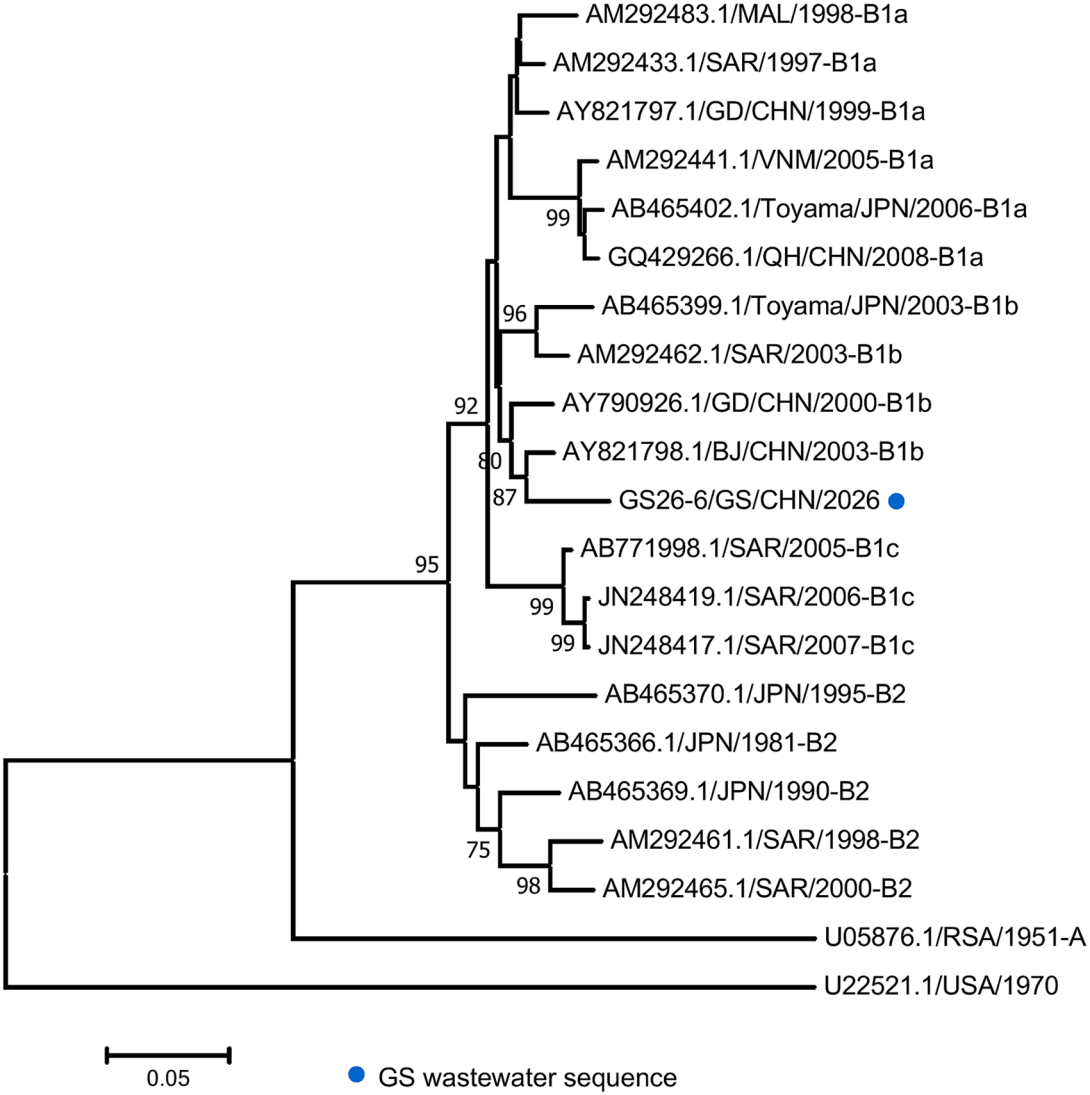
Neighbor-joining phylogenetic tree based on partial VP1 gene (capsid) sequences of coxsackievirus A16. The sequence derived from Gansu wastewater sample GS26-6 is indicated by a blue dot. The remaining 20 sequences comprised 19 representative CV-A16 genotype reference sequences and one EV-A71 outgroup sequence (U22521.1), all retrieved from GenBank. Bootstrap values ≥70% are indicated.

A total of 5 sequences spanning the CV-A10 VP1 region were obtained and included in a phylogenetic analysis with CV-A10 sequences recently reported in China. The 5 wastewater-derived Gansu sequences were located on closely related branches but did not form a monophyletic clade. GS24-1/GS/CHN/2024 and GS25-5/GS/CHN/2025 clustered as sister taxa, with 86% bootstrap support. GS26-4/GS/CHN/2026 clustered with the Jiangxi reference strain PP862448.1/JX/CHN/2023, with 75% bootstrap support. GS25-6/GS/CHN/2025 and GS26-2/GS/CHN/2026 further clustered with this clade, with 80% bootstrap support at the relevant internal node (Figure 7). These results indicate that the CV-A10 sequences detected in Gansu wastewater were genetically closely related and showed phylogenetic relationships with reference sequences from other regions of China.

**Figure 7:**
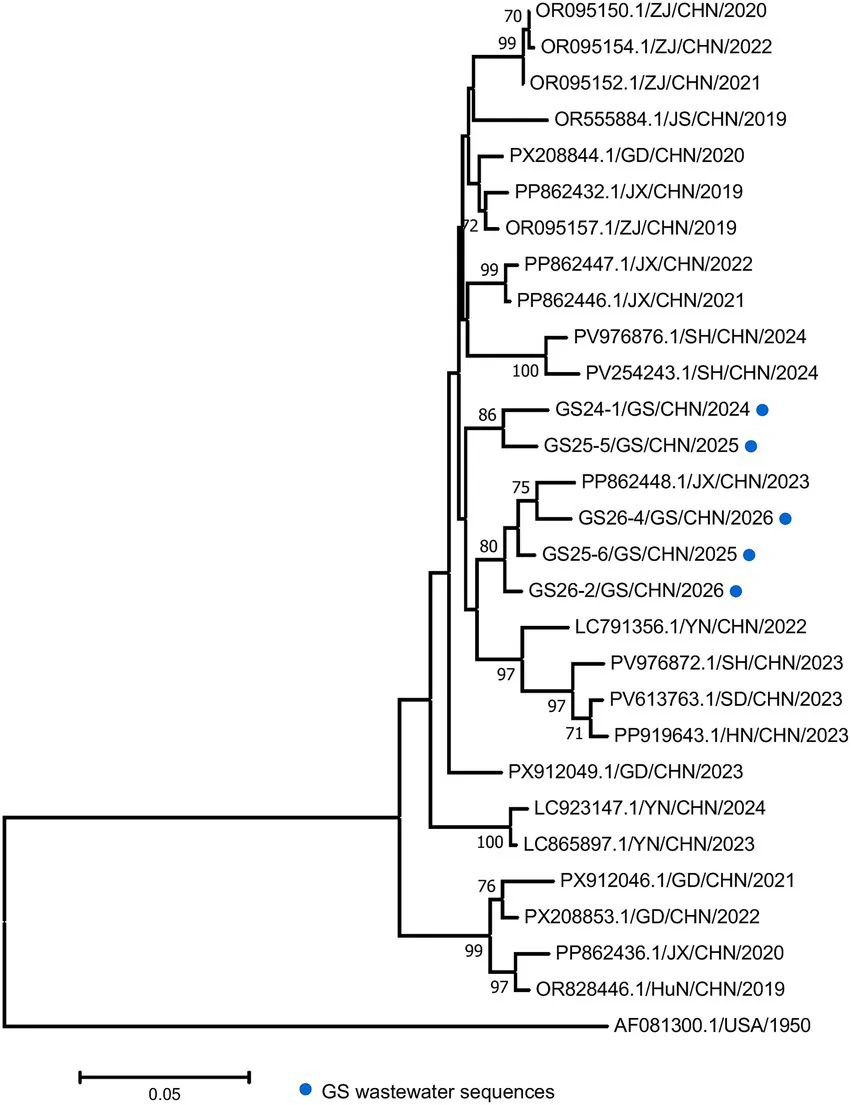
Neighbor-joining phylogenetic tree based on partial VP1 gene (capsid) sequences of coxsackievirus A10. The sequences derived from Gansu wastewater samples are indicated by blue dots. The remaining sequences were obtained from reference strains retrieved from GenBank. Bootstrap values ≥70% are indicated.

The PCA highlighted variation in enterovirus composition among samples collected during the three sampling campaigns (Figure 8). Together, PC1 (20.0%) and PC2 (16.4%) represented 36.4% of the total variance. This distribution is presented as an exploratory visualization because sampling year coincided with collection month and between-group differences were not evaluated using an inferential test.

**Figure 8:**
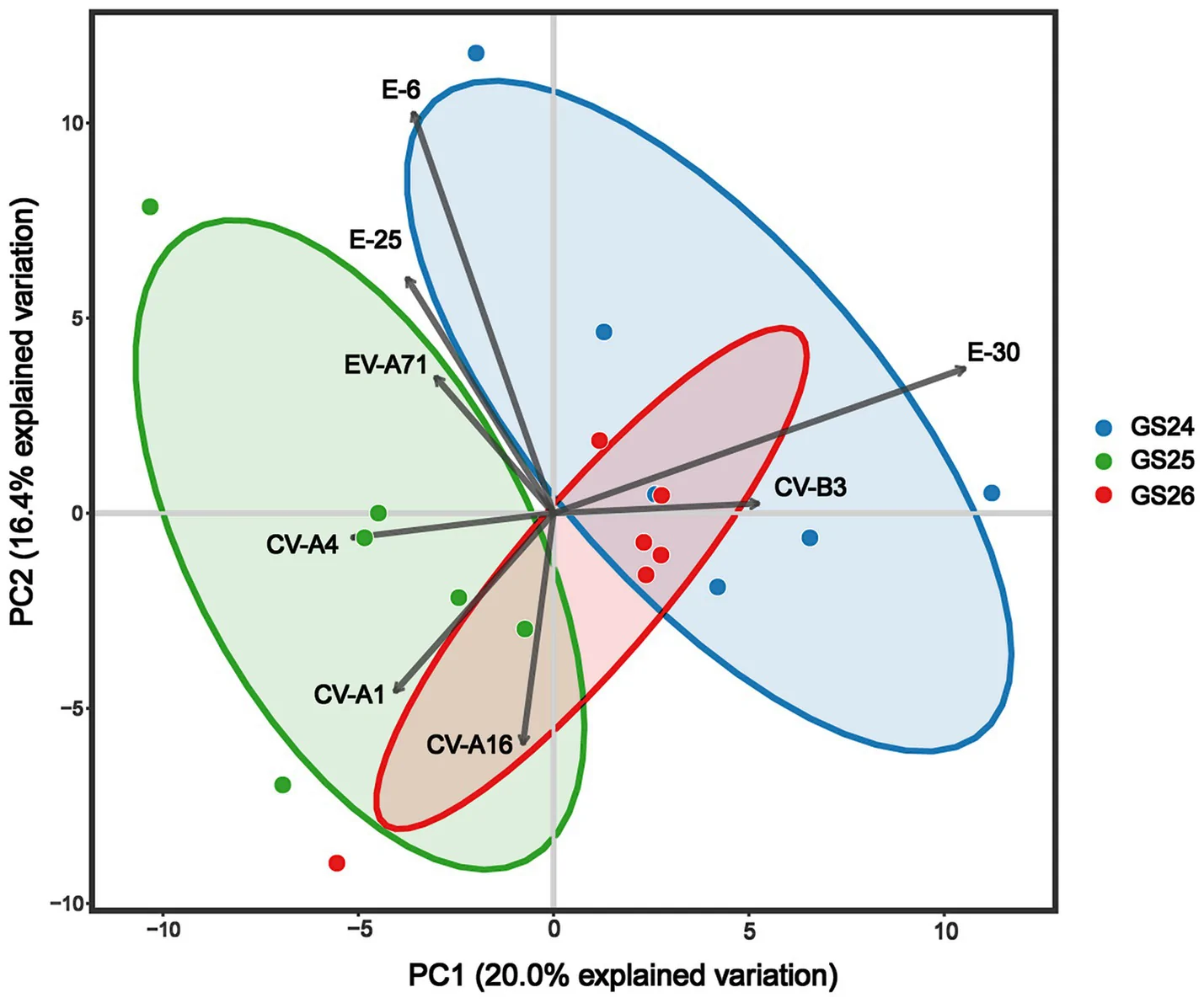
PCA plot of enterovirus composition across three wastewater sample groups. Arrows denote types: their orientation shows the contribution to the principal components, whereas their length represents the magnitude of the corresponding loading.

## Discussion

Wastewater metagenomic alpha diversity exhibited variation across the three sampling years. Samples were collected in July 2024, June 2025, and March 2026; therefore, sampling year was intrinsically linked to sampling month and season in this study. The composition of sampling sites also varied across years: Hezuo and Zhangye were each represented by two samples in 2024 and 2025, respectively, whereas Dingxi was included only in 2026. Statistical analysis revealed a significant negative correlation between the Simpson index and sampling year, whereas the Shannon index showed a negative but nonsignificant correlation, suggesting that the two diversity metrics captured different aspects of community variation. Overall, the observed changes in alpha diversity may reflect the combined effects of annual, seasonal, and spatial factors. Environmental conditions may have contributed to these between-year differences. Previous research on influent wastewater bacterial communities has demonstrated seasonal structuring associated primarily with wastewater temperature and, in some settings, with precipitation, wastewater flow, and physicochemical variables (LaMartina et al., 2021). These variables should therefore be regarded as plausible confounders of the observed decrease in the present study. Changes in the size, mobility, dietary habits, and infection burden of the contributing population could also plausibly alter the biological inputs into wastewater; however, corresponding demographic data were not available, and their effects could not be evaluated.

Technical factors may also have influenced the observed metagenomic profiles. Although clarification and 0.22 μm filtration were used to reduce intact bacterial cells and larger particulate material, filtration alone cannot eliminate extracellular or particle-protected bacterial nucleic acids remaining in a complex wastewater matrix. In addition, the MgCl_2_-assisted membrane adsorption and elution procedure was used to concentrate virus-containing material but was not a virus-specific affinity-purification step. Most importantly, total nucleic acid was extracted without DNase treatment, allowing residual bacterial DNA and other unprotected DNA to enter the sequencing libraries. These factors likely contributed to the predominance of bacterial reads (>97%) despite viral enrichment, indicating that the procedure concentrated virus-containing wastewater material rather than producing a purified virome. The persistence of bacterial sequences following virion-enrichment procedures has also been reported in sewage metagenomic studies (Cantalupo et al., 2011). Pretreatment with DNase-based enzymes or Benzonase-containing nuclease cocktails before capsid disruption and nucleic acid extraction has been incorporated into virome-preparation workflows to remove non-particle-protected nucleic acids (Cantalupo et al., 2011; Conceicao-Neto et al., 2015; Brinkman et al., 2018). Database-dependent classification represents an additional source of uncertainty because prophage-related sequences and the incomplete representation of environmental microorganisms and viruses in reference databases may affect individual taxonomic assignments (Cantalupo et al., 2011; Roux et al., 2015; Paez-Espino et al., 2016). Furthermore, while the same general experimental workflow was used across the 3 years, unmeasured variation in transport duration, nucleic acid recovery, or sample-processing and sequencing batches could not be separated from sampling year. Kraken2 was unable to classify 56.42–74.74% of the metagenomic reads at the genus level. This unclassified fraction may contain viral sequences that are highly divergent from existing references or poorly represented in current databases—potentially constituting viral “dark matter”—but may also include sequences of nonviral origin and reads containing insufficient information for reliable classification.

Among the bacteriophage communities, taxa represented by the family *Fiersviridae* exhibited high abundance and stability across different time points. As key members of the *Fiersviridae* family, F-specific RNA phages stably exist in aquatic environments as potential viral indicators of water pollution (Hata et al., 2016). The persistent presence of the families *Astroviridae* and *Picornaviridae* (including enteroviruses and kobuviruses) may reflect widespread community shedding. Conversely, the family *Reoviridae* and the family *Hepeviridae* consistently remained at extremely low background levels across all temporal cohorts. Because population-level data on virus-specific infection prevalence and vaccination coverage were unavailable, the low relative abundances observed in wastewater could not be reliably attributed to these factors. Furthermore, given the potential detection biases inherent in metagenomic sequencing of complex wastewater samples, these findings should be interpreted cautiously, as the observed low abundances may partly reflect methodological limitations rather than genuinely low viral abundance. Therefore, in future surveillance, supplementary validation combining highly specific targeted enrichment probes or digital PCR technologies may be required for these low-abundance yet high-risk target viruses, to avoid missing potential sporadic sources of infection.

The consistently high abundance of plant-associated viruses in wastewater, particularly the genera *Tobamovirus* and *Potexvirus*, is not surprising. Previous studies have demonstrated that plant viruses dominate the viral communities in human fecal samples and are widespread across aquatic biomes (Zhang et al., 2006). Specifically, the genus *Tobamovirus* primarily originates from the daily human consumption of infected plants and their processed products, which pass through the digestive tract and are excreted in large quantities in feces. Although members of the genus *Tobamovirus* exhibit strict plant-host specificity, a case–control study reported associations between the detection of pepper mild mottle virus in human stool and specific immune responses and clinical symptoms, including fever, abdominal pain, and pruritus; however, causality was not established (Colson et al., 2010; Editors, 2026). Given its exceptionally high environmental abundance and potential relevance to human health, pepper mild mottle virus warrants consideration for inclusion in environmental surveillance systems. Beyond this, pepper mild mottle virus has been widely validated as an ideal environmental indicator for evaluating the viral removal efficacy of wastewater treatment processes, such as conventional activated sludge and membrane bioreactors (Canh et al., 2025). The high relative abundance of members of the genus *Potexvirus* in this study is noteworthy. Members of this genus primarily infect herbaceous plants, including many economically important crops. Potato virus X, a representative member of the genus and a member of the species *Potexvirus ecspotati*, is an important pathogen of potato plants (Verchot, 2022). The frequent detection of potexviruses in wastewater may be associated with the agricultural and dietary context of Gansu Province, where potatoes are extensively cultivated and commonly consumed. Viral particles or nucleic acids originating from infected plant materials may enter municipal wastewater through food processing, food-waste disposal, and excretion following consumption. Sewage pipelines frequently harbor substantial insect populations (Cantalupo et al., 2011), which explains the detection of entomopathogenic viral genera such as *Alphanodavirus* and *Betabaculovirus*. In nature, these viruses play an ecological role in regulating insect populations, and certain groups, like baculoviruses, have been developed as microbial control agents (Lacey et al., 2015). Among the 9 human-associated viral agents analyzed, astroviruses exhibited the highest relative abundance. Surveillance data revealed the stable presence of the genus *Hepatovirus* across the three-year study. As the sole human-infecting member within the genus *Hepatovirus*, hepatitis A virus (HAV) exhibits significant regional endemic characteristics in China. Previous data indicated that the incidence of HAV infection in Gansu Province was among the highest in China, exceeded only by the corresponding rates in Sichuan, the Xinjiang Uygur Autonomous Region, Yunnan, and Henan (Xu, Y., et al., 2025). This high endemicity underscores the urgency of implementing more rigorous epidemiological surveillance in this region and highlights the critical need to synergistically promote targeted vaccination programs and public health education to effectively curtail the regional transmission of HAV (Franco et al., 2012).

The high detection frequency of astroviruses in this study is highly consistent with the results of previous wastewater sequencing in the Xinjiang Uygur Autonomous Region (Yang et al., 2021). HAstVs are increasingly recognized as potential causes of severe systemic and neurological infections (Wang et al., 2025). HAstV-1, HAstV-2, and HAstV-4 belong to classical human astroviruses classified within the species *Mamastrovirus hominis* and are common causes of viral gastroenteritis in infants, elderly individuals, and immunocompromised populations. Among them, HAstV-1, as the most common circulating genotype globally, occupied a dominant position in this wastewater surveillance. This highlights the potential value of wastewater surveillance for monitoring astrovirus epidemiological trends. MLB viruses are among the more recently discovered human astroviruses, and the detection of human astrovirus MLB2 in wastewater warrants further surveillance (Finkbeiner et al., 2009). Recent studies have shown that MLB2 viruses, which are frequently detected in fecal samples, have the potential for extraintestinal dissemination and may represent an underrecognized cause of central nervous system infections among both immunocompetent and immunocompromised individuals (Cordey et al., 2016); therefore, the environmental detection of MLB2 highlights the potential value of integrating environmental surveillance with clinical monitoring.

Targeted sequencing revealed the unique characteristics of the local environmental EV composition profile. CV-A6 was the predominant enterovirus type within the *Enterovirus alphacoxsackie* detected in wastewater, with a relative abundance higher than that of coxsackievirus A16 (CV-A16) and EV-A71. EV-A71 (belonging to the *Enterovirus alphacoxsackie* group), which previously caused numerous severe cases of HFMD (Li et al., 2025), was detected at a lower abundance in the wastewater. The lower relative read abundance of EV-A71 may reflect a combination of methodological, environmental, and epidemiological factors; changes in community circulation cannot be inferred without supporting clinical, vaccination coverage, and epidemiological data.

Coxsackievirus A19, a fastidious virus belonging to the *Enterovirus coxsackiepol* group, may be underdetected in routine clinical surveillance in China because such surveillance relies heavily on cell culture-based detection systems (Yi et al., 2022). Enterovirus B83 was also detected in the wastewater; this virus is a non-polio enterovirus closely associated with acute viral myocarditis and acute flaccid paralysis (Xiao et al., 2020; Song et al., 2023). The predominance of echovirus 11 in environmental wastewater surveillance in Gansu Province was consistent with previous findings from Shandong Province during 2015–2017 (Zhao et al., 2020).

Following the introduction of the EV-A71 vaccine in China, the proportion of HFMD outbreaks attributed to CV-A16 increased (Liu et al., 2025). For CV-A16, previous studies based on complete VP1 gene (capsid) sequences or whole-genome sequences have demonstrated that the B1a and B1b subgenotypes cocirculated in mainland China over an extended period, with B1b maintaining a higher proportion across multiple regions over a longer timescale (Chen et al., 2013). The B1b-related sequences detected in the wastewater in this study align with the historical transmission background of this lineage in China. However, only a single sequence matching the CV-A16 VP1 region was recovered in this study from a wastewater sample collected in Dingxi City in March 2026. Therefore, these data are insufficient to determine whether B1b is currently the predominant lineage in Gansu Province or to estimate the prevalence of CV-A16 infection in the population. Notably, recent surveillance in Guangdong and Hangzhou revealed that the B1c lineage has increased markedly since 2023, becoming the dominant or codominant lineage in those regions by 2024, suggesting that the lineage composition of CV-A16 may vary across different regions and time periods (Xu, Q., et al., 2025; Zeng et al., 2025).

Among the most common viruses causing HFMD, the public health burden of CV-A10 may be underestimated because its incidence is lower than that of other common causative viruses, although CV-A10-associated cases tend to be more severe (Bian et al., 2019). The CV-A10 sequences detected in Gansu wastewater between 2024 and 2026 clustered within a relatively confined region of the phylogenetic tree, suggesting that CV-A10 lineages with similar genetic backgrounds may have circulated widely in this area. In particular, GS24-1, detected in 2024, and GS25-5, detected in 2025, formed a well-supported clade, indicating that they shared a relatively recent common ancestor and potentially reflecting the persistence of a related viral lineage across different years. In addition, GS26-4, detected in 2026, clustered with the 2023 Jiangxi reference strain PP862448.1, indicating a close genetic relationship between the Gansu wastewater-derived sequences and strains circulating in other regions. This finding may reflect the circulation of related CV-A10 lineages across different geographical regions.

The findings of this study have several pathogen-specific implications for public health surveillance in Gansu Province. Given the current absence of approved vaccines or specific antiviral therapies for preventing or treating HAstV infection (Wang et al., 2026), its persistent detection in wastewater underscores the need for local public health authorities to strengthen case-based and wastewater surveillance, improve drinking-water safety and food hygiene, reinforce infection-control measures in childcare facilities, and provide targeted health education. Although positive wastewater signals do not, by themselves, indicate an increase in clinical incidence, echovirus 11 accounted for a substantial proportion of the detected enterovirus signals and may cause severe neonatal infections (Ying et al., 2024). In addition, CV-A6 emerged as the predominant type within *Enterovirus alphacoxsackie*, with a higher relative abundance than CV-A16 and EV-A71, two types traditionally associated with HFMD (Bian et al., 2015). Routine wastewater sampling across the province is therefore recommended during locally defined enterovirus epidemic seasons, with reverse transcription quantitative PCR (RT-qPCR) surveillance coupled with VP1-region or whole-genome sequencing for viral typing and lineage assignment. Concurrent positive signals at multiple surveillance sites should prompt enhanced enterovirus testing of specimens from neonatal infection cases and timely adjustment of HFMD prevention and control strategies according to changes in wastewater-detected viral types. Further recommendations include strengthening etiological sampling and testing among HFMD cases, investigating clusters in childcare facilities and schools, providing targeted health and hygiene education, assessing EV-A71 vaccination coverage, and maintaining surveillance for enterovirus types other than EV-A71.

The methodological framework applied in this study is broadly transferable to other regions. The combination of untargeted metagenomic sequencing for broad viral signal discovery, targeted sequencing for sensitive detection and characterization of selected pathogens, and wastewater as a community-level sampling matrix provides a flexible approach for multi-pathogen surveillance. Its implementation should be adapted to local environmental conditions, population structures, and wastewater infrastructure through appropriate catchment mapping and local validation of sampling designs and analytical thresholds. Influent from wastewater treatment plants may be used in regions with centralized sewer systems, whereas representative community drains or discharge points may be selected in areas with incomplete or decentralized sewer coverage. Routine sentinel surveillance should standardize sampling sites, sampling frequency, sample volume, transport time, laboratory procedures, and the collection of environmental and catchment-level metadata, including temperature, precipitation, wastewater flow, sewer configuration, and population coverage. Wastewater flow and appropriate fecal-strength indicators may also be incorporated to improve normalization and comparability across sites. Unexpected detections or signals exceeding locally established thresholds should prompt repeat sampling and orthogonal confirmation using RT-qPCR, digital PCR, or targeted sequencing. Confirmed wastewater signals should subsequently be interpreted together with clinical surveillance data for HFMD, gastroenteritis, hepatitis A, acute flaccid paralysis, and other relevant diseases or syndromes. This integrated workflow therefore provides an adaptable surveillance strategy that can be implemented across diverse regional settings.

This exploratory study has several limitations. First, because samples were collected in July 2024, June 2025, and March 2026, sampling year was completely confounded with season. The absence of corresponding environmental and demographic covariates further prevented separation of seasonal, environmental, demographic, and site-composition effects. Second, the viral enrichment and total-nucleic-acid extraction procedures require further optimization. The high bacterial background reduced the effective viral sequencing depth and may have decreased sensitivity for low-abundance targets. Third, the study did not include matrix-spike or process controls for evaluating viral recovery, independent nucleic acid extraction or library-preparation replicates, confirmatory RT-qPCR, digital PCR, or Sanger sequencing, or systematic cross-validation of Kraken2 assignments using an alternative classifier. Fourth, taxonomic assignments depended on existing reference databases. Unclassified or ambiguous sequences may represent poorly characterized viruses, nonviral sequences, or degraded genetic material, whereas highly divergent or novel viruses may have been missed or misclassified. Fifth, read-based relative abundance is affected by nucleic acid recovery, genome length, library preparation, sequencing depth, and compositional effects and therefore cannot be translated directly into population-level infection prevalence or clinical incidence. Finally, the absence of contemporaneous clinical, vaccination, population-denominator, wastewater-flow, and quantitative molecular data limited the epidemiological interpretation of the wastewater signals. Future studies should address these limitations through seasonally matched longitudinal sampling, standardized collection of environmental and catchment-level metadata, appropriate process controls and technical replicates, improved viral enrichment and nuclease treatment, orthogonal molecular and bioinformatic validation, and integration with contemporaneous clinical, vaccination, population, and wastewater-flow data.

## Data Availability

The datasets presented in this study can be found in online repositories. The names of the repository/repositories and accession number(s) can be found in the article/Supplementary material.

## Footnotes

1 https://www.bioinformatics.babraham.ac.uk/projects/fastqc/.

2 http://huttenhower.sph.harvard.edu/kneaddata.

3 https://blast.ncbi.nlm.nih.gov/.

4 http://www.r-project.org/.

5 https://www.genome.jp/virushostdb/.
