# Supplementary material for: High-throughput sequencing reveals the virome characteristics of wastewater in Gansu Province, China from 2024 to 2026
Source: Front Microbiol. 2026 Sep 22;17:1966428. doi: 10.3389/fmicb.2026.1966428 (PMC13638646; doi:10.3389/fmicb.2026.1966428)
Supplement: Supplementary file 1 [file Table_1.DOCX]

Data are deposited in National Microbiology Data Center (NMDC) with accession numbers NMDC40127272、NMDC40127273、NMDC40127274、NMDC40127275、NMDC40127276、NMDC40127277、NMDC40127278、NMDC40127279、NMDC40127280、NMDC40127281、NMDC40127282、NMDC40127283、NMDC40127284、NMDC40127285、NMDC40127286、NMDC40127287、NMDC40127288、NMDC40127289、NMDC40127290、NMDC40127291、NMDC40127292、NMDC40127293、NMDC40127294、NMDC40127295、NMDC40127296、NMDC40127297、NMDC40127298、NMDC40127299、NMDC40127300、NMDC40127301、NMDC40127302、NMDC40127303、NMDC40127304、NMDC40127305、NMDC40127306、NMDC40127307 (https://nmdc.cn/resource/search).

**Supplementary Table 1.** Proportions of metagenomic reads remaining unclassified at the genus level after Kraken2 classification.

| Sample | Unclassified (%) | Sample | Unclassified (%) | Sample | Unclassified (%) |
| --- | --- | --- | --- | --- | --- |
| GS24-1 | 68.67 | GS25-1 | 60.42 | GS26-1 | 74.74 |
| GS24-2 | 74.04 | GS25-2 | 67.75 | GS26-2 | 73.98 |
| GS24-3 | 72.68 | GS25-3 | 67.83 | GS26-3 | 69.58 |
| GS24-4 | 68.77 | GS25-4 | 56.42 | GS26-4 | 69.02 |
| GS24-5 | 70.15 | GS25-5 | 65.87 | GS26-5 | 74.62 |
| GS24-6 | 74.36 | GS25-6 | 65.08 | GS26-6 | 67.35 |

**Supplementary Table 2.** Full names of type abbreviations used in Figure 5.

| Abbreviation | Full Name | Abbreviation | Full Name |
| --- | --- | --- | --- |
| CV-A2 | Coxsackievirus A2 | E-3 | Echovirus 3 |
| CV-A4 | Coxsackievirus A4 | E-6 | Echovirus 6 |
| CV-A5 | Coxsackievirus A5 | E-11 | Echovirus 11 |
| CV-A6 | Coxsackievirus A6 | E-25 | Echovirus 25 |
| CV-A10 | Coxsackievirus A10 | E-29 | Echovirus 29 |
| CV-A14 | Coxsackievirus A14 | E-30 | Echovirus 30 |
| CV-A16 | Coxsackievirus A16 | EV-B83 | Enterovirus B83 |
| EV-A71 | Enterovirus A71 | CV-A1 | Coxsackievirus A1 |
| CV-A9 | Coxsackievirus A9 | CV-A13 | Coxsackievirus A13 |
| CV-B2 | Coxsackievirus B2 | CV-A19 | Coxsackievirus A19 |
| CV-B3 | Coxsackievirus B3 | CV-A22 | Coxsackievirus A22 |
| CV-B4 | Coxsackievirus B4 | CV-A24 | Coxsackievirus A24 |
| CV-B5 | Coxsackievirus B5 | PV-3 | Poliovirus 3 |
